# Structural and Chemical Characterization of Hardwood from Tree Species with Applications as Bioenergy Feedstocks

**DOI:** 10.1371/journal.pone.0052820

**Published:** 2012-12-28

**Authors:** Özgül Persil Çetinkol, Andreia M. Smith-Moritz, Gang Cheng, Jeemeng Lao, Anthe George, Kunlun Hong, Robert Henry, Blake A. Simmons, Joshua L. Heazlewood, Bradley M. Holmes

**Affiliations:** 1 Joint BioEnergy Institute, Lawrence Berkeley National Laboratory, Berkeley, California, United States of America; 2 Physical Biosciences Division, Lawrence Berkeley National Laboratory, Berkeley, California, United States of America; 3 Joint BioEnergy Institute, Sandia National Laboratory, Livermore, California, United States of America; 4 Center for Nanophase Material Sciences, Oak Ridge National Laboratory, Oak Ridge, Tennessee, United States of America; 5 Queensland Alliance for Agriculture and Food Innovation, The University of Queensland, St Lucia, Queensland, Australia; Iowa State University, United States of America

## Abstract

Eucalypt species are a group of flowering trees widely used in pulp production for paper manufacture. For several decades, the wood pulp industry has focused research and development efforts on improving yields, growth rates and pulp quality through breeding and the genetic improvement of key tree species. Recently, this focus has shifted from the production of high quality pulps to the investigation of the use of eucalypts as feedstocks for biofuel production. Here the structure and chemical composition of the heartwood and sapwood of *Eucalyptus dunnii, E. globulus, E. pillularis, E. urophylla,* an *E. urophylla-E. grandis* cross, *Corymbia citriodora* ssp. *variegata*, and *Acacia mangium* were compared using nuclear magnetic resonance spectroscopy (NMR), X-ray diffraction (XRD) and biochemical composition analysis. Some trends relating to these compositions were also identified by Fourier transform near infrared (FT-NIR) spectroscopy. These results will serve as a foundation for a more comprehensive database of wood properties that will help develop criteria for the selection of tree species for use as biorefinery feedstocks.

## Introduction

The selection and development of a wide range of sustainable biomass feedstocks is critical for the establishment of a viable biorefinery. There are several renewable resources capable of providing sufficient quantities of lignocellulose annually for the production of biofuels and chemicals, including crops like *Miscanthus giganteus* and switchgrass, agricultural residues, and woody biomass derived from the forestry industry. It will be necessary to adopt a diversified regional approach to maximize yields, taking advantage of the natural pairings of climate, water resources, soil type, infrastructure, and geography.

Eucalypts are the most widely planted hardwoods, representing approximately 8% of all productive planted forests. They are grown in tropical and subtropical regions of Asia, Australia, Africa and South America, as well as in temperate regions in Europe, South and North America, Australia and Africa. Eucalypts are now placed in several genera including the widespread *Corymbia* and *Eucalyptus* genera [1]. Together with *Acacia* spp., eucalypts cover over 19.3×10^6^ hectares and are among the fastest growing hardwood trees, with an average yield (over 10–20 year rotations) of 5–10 m^3^·ha^−1^·yr^−1^ in drier climates, and up to 30 m^3^·ha^−1^·yr^−1^ and greater in regions with high annual rainfall [2]. These evergreen trees can be harvested year round and, as they can be grown on upland landscapes, reduce the pressure on environmentally sensitive areas.

To date, the majority of literature on *Eucalyptus* and *Acacia* spp. has focused on its use as a source of pulps for the manufacture of high quality paper and tissue [3], [4], [5]. In the area of renewable energy, eucalypts have been studied as a source of charcoal for the production of “green steel” [6], [7], [8], and more recently, for the production of ethanol after pretreatment and enzymatic hydrolysis [9], [10], [11], [12], [13]. A number of studies employing a variety of feedstocks have demonstrated the relationship between the composition and structure of the biomass and improvements in saccharification. These have included alterations in lignin content in alfalfa, reed canarygrass and switchgrass [14]; lignin content and monomer composition in Arabidopsis [15]; lignin monomer ratios in switchgrass [16]; and cellulose crystallinity in avicel samples [17].

Given the natural and geographical diversity present in hardwoods, it is necessary to know how composition varies between species and how it is influenced by environmental factors rapidly. This understanding will aid in the selection of species most suited for conversion into biofuels. The heartwood and sapwood of six eucalypts and a widely planted tropical wattle, *Acacia mangium*, were analyzed to establish a baseline in the variation of their polysaccharide and lignin composition, chemical linkages, and cellulose crystallinity.

## Materials and Methods

### Plant Material and Processing

The heartwood and sapwood of Eucalyptus dunnii, Eucalyptus globulus, Eucalyptus pillularis, Eucalyptus urophylla, Eucalyptus urophylla-Eucalyptus grandis cross, Corymbia citriodora subsp. variegata (CCV) and Acacia mangium were sampled in 2009 from a common field planting in Grafton, New South Wales, Australia. No specific permits were required for the described field studies. The hardwood samples were obtained from trial plantings by Forests NSW (Department of Primary Industries, New South Wales) and were not collected from wild trees and do not involve endangered or protected species. The samples were supplied by Michael Henson a tree breeder with Forests NSW. These species were chosen to represent the major commercial species planted in forests in Australia and globally. Heartwood (HW) and sapwood (SW) samples from single trees of the selected species were Wiley® milled to pass through a 40 mesh screen and Soxhlet extracted with water overnight and ethanol for 4 to 6 hours. The extracted biomass was dried at 40°C in a vacuum oven and stored at 4°C.

### Nuclear Magnetic Resonance (NMR) Spectroscopy

Extracted biomass was cryo-milled in a SpexSamplePrep 6770 Freezer/Mill (SPEX CertiPrep, Metuchen, NJ) for 12 cycles in total. Each cycle consisted of two minutes of grinding time and two minutes of cool down time. The cryo-milled samples (∼55 mg) were then placed in NMR tubes (542-PP-7, Wilmad LabGlass, Buena, NJ) with 600 µL of 99.9% dimethyl sulfoxide-d6 (DMSO-d6, Cambridge Isotope Laboratories, Inc. Andover, MA). The samples were sealed and sonicated, until homogenous, in a Branson 2510 tabletop cleaner (Branson Ultrasonic Corporation, Danburt, CT). The temperature of the bath was maintained below 45°C. 1H and HSQC (hsqcetgpsi2, td = 1 k, ns = 300, ds = 16, number of increments = 256, d1 = 1 s) spectra were acquired at 298 K using a Bruker (Billerica, MA) Avance–600 MHz equipped with a cryo-probe. The central DMSO peak was used as an internal reference; δH 2.49 and δC 39.5 ppm. The software program Topspin™ (Bruker BioSpin) was used for processing and analysis of the data.

### X-ray Diffraction (XRD)

X-ray diffraction measurements were performed with the PANalytical Powder Diffractometer with a Cu-Kα source (λ = 1.54 Å), operated at 45 KV and 40 mA. The scans were collected between 2Θ = 5° to 40°, with a step size of 0.017°. The crystallinity of a sample, expressed as the percentage of crystalline cellulose in the whole biomass sample, and can be estimated by using the empirical equation: CrI = (I*_total_*-I*_amorphous_*/I*_total_*) where I*_total_* and I*_amorphous_* correspond to the scattering intensity of the whole sample and the amorphous materials in the sample respectively. Two measures of I*_total_* and I*_amorphous_* were used to estimate the crystallinity of the samples; 1) the Segal method, with I*_total_* and I*_amorphous_* being equal to the intensity of diffraction at the main peak and the minimum intensity between the main and secondary peaks, respectively and 2) the Ruland-Vonk method where I*_total_* being derived from the sample, and I*_amorphous_* from an amorphous standard, in this case pine derived kraft lignin (Mead West Vaco**,** Richmond, VA). Overall, this equates I*_total_* and I*_amorphous_* to the area under the scattering profile of the samples and lignin standard respectively, where the scattering profile from lignin was adjusted such that the background scattering matches that of the samples [18].

### Two Stage Acid Hydrolysis

Total carbohydrate and lignin content of each extracted biomass sample was measured in triplicate using the National Renewable Energy Laboratory procedure TP-510-42618 [19]. In summary, a HB43-S Halogen Moisture Analyzer (Mettler-Toledo) was used to determine the moisture content (Table S1) of wood samples. Around 300 mg of extracted biomass was placed in 140 ml pressure tubes (Ace Glass Inc, # 8648-30 Vineland, NJ) and mixed with 3 ml of 72% sulfuric acid (H_2_SO_4_; Fisher Scientific). Samples were incubated at 30°C for one hour and macerated with glass rods every five minutes after which 84 g of H_2_O was added to each tube. The pressure tubes were then sealed, mixed well by inversion and autoclaved for one hour at 121°C using a liquid cycle. The samples were then cooled to room temperature and filtered through pre-weighed glass fiber filters (Whatman GA) and dried at 105°C. A sample of the filtrate was taken from each digestion for total carbohydrate analysis by HPAEC and acid soluble lignin analysis. The retentate was further washed with 3×80 ml of water to remove any residual acid. The acid insoluble lignin content was measured as the oven dried weight of the retentate. The ash content of the biomass could not be measureable due to relative low reported content of less than 0.3% [9], [20], [21], [22], [23]. Acid soluble lignin content was calculated by using UV absorbance of the filtrate at 205 nm (Shimadzu UV-2401, Columbia, MD). An extinction coefficient of 110 l·g^−1^·cm^−1^ was used to determine the lignin concentration [24].

### Monosaccharide Composition Analysis

The matrix polysaccharide content was measured in triplicate using extracted biomass samples [25]. Briefly, a total of approximately 5 mg of extracted biomass was incubated in 1 ml of 2M (15% ^v^/_v_) trifluoroacetic acid (TFA, Sigma-Aldrich) at 120°C for an hour in 2 ml screw cap tubes. The tubes cooled down to room temperature and the samples dried in a vacuum concentrator and weighed to obtain alcohol insoluble residue (AIR) mass. Afterwards, 1 ml of H_2_O was added to each tube, and tubes were shaken at room temperature for 30 min to dissolve soluble sugars. Samples were centrifuged for 10 min at 20,000 *g* resulting in a soluble and insoluble fraction. A 10 µL aliquot from the soluble fraction of each sample was diluted 1∶50 with water and analyzed for monomeric sugars using HPAEC. Cellulose content was determined from the 20,000 *g* TFA insoluble fraction (pellet), using the Updegraff method [26]. Briefly, the insoluble pellet was washed with H_2_O and acetone and dried overnight in a vacuum concentrator. The dried pellet was resuspended in 67% H_2_SO_4_ shaking at room temperature for 1 hour. The sample was clarified at 20,000 *g* and diluted with 0.2% anthrone (Sigma-Aldrich) in concentrated H_2_SO_4_ and incubated in boiling water for 5 min. Glucose concentration was measured using a spectrophotometer at λ = 620 nm against a standard curve prepared with Avicel (Sigma-Aldrich). The hemicellulose composition and glucose derived cellulose content were calculated based on the AIR mass of each sample.

### High Performance Anion Exchange Chromatography

Monosaccharide composition was used to calculate the total carbohydrate content from H_2_SO_4_ derived filtrate and TFA derived matrix polysaccharide digestion of extracted. All samples were measured in triplicate using High Performance Anion Exchange Chromatography with Pulsed-Amperometric Detection (HPAEC-PAD) on a Dionex DX600. Separation was achieved with a CarboPac PA-20 analytical column (3×150 mm) and a CarboPac PA-20 guard column (3×30 mm) (Dionex, Sunnyvale, CA), at a temperature of 30°C with an eluent flow rate of 0.4 mL/min. The following method was employed, 12 min elution with 14 mM NaOH; 5 min ramp to 450 mM NaOH; 10 min elution with 450 mM NaOH; 10 min equilibration with 14 mM NaOH. In order to determine the mannose and xylose content for each sample the following modified method was applied. A CarboPac SA-10 analytical column (3×150 mm) at a temperature of 30°C with an eluent flow rate of 0.4 mL/min employing the following gradient: 25 min elution with 1 mM NaOH followed by a 15 min ramp to 10 mM NaOH. External standards of fucose, rhamnose, arabinose, mannose, galactose, glucose, xylose, galacturonic acid and glucuronic acid were used to determine the concentration.

### Fourier Transform Near Infrared Spectroscopy (FT-NIR)

A MPA FT-NIR Spectrometer (Bruker Optics) was used to obtain spectra from each biomass sample. Spectral absorbance for 6–8 sample replicates of each species for all of heartwood and sapwood samples, covering a range from 12,000 to 3,800 cm^−1^, was taken at a spectral resolution of 8 cm^−1^. Spectra were collected in diffuse reflectance mode. A total of 32 scans were taken for each sample and co-added to give the final spectra. Preprocessing of the absorption spectra was done using Opus software (Bruker Optics). Absorption spectra were cropped from 9,000 to 4,000 cm^−1^, smoothed using a Savitzky–Golay filter with 25 points, and baseline corrected. Statistical analysis was carried using Matlab (Mathworks). After pre-processing of the spectra, the data set was area-normalized then mean centered. Principal component analysis (PCA) was used for data compression [27]. To compare inter-species (group) variability with intra-species variability (biological and technical), Canonical Variate Analysis (CVA) was then performed on the PC scores that accounted for 95% of the variation across all samples (heartwood = 6 pc scores, sapwood = 11 pc scores) [27].

## Results and Discussion

An important aspect to the development of feedstocks is knowledge regarding what specific structural and biochemical components in hardwoods play significant roles in biofuels production. Consequently, detailed studies on a wide variety of feedstocks employing various analytical techniques are required to establish a framework the burgeoning industry. Several important steps must initially be optimized including the structural loosening of cell wall by pretreatment for enhanced enzymes accessibility and digestibility, followed by hydrolysis of cell wall to fermentable sugars then finally fermentation of sugars into fuels [28], [29]. Each step is predicated on the previous process therefore to properly begin this avenue of research a complete compositional analysis is required prior to the assessment of biomass saccharification. To the best of our knowledge, these basic issues have not been explored in these hardwood species and consequently initial analyses of structure and composition are required.

### Analysis by 2D Nuclear Magnetic Resonance Spectroscopy

**Figure 1:**
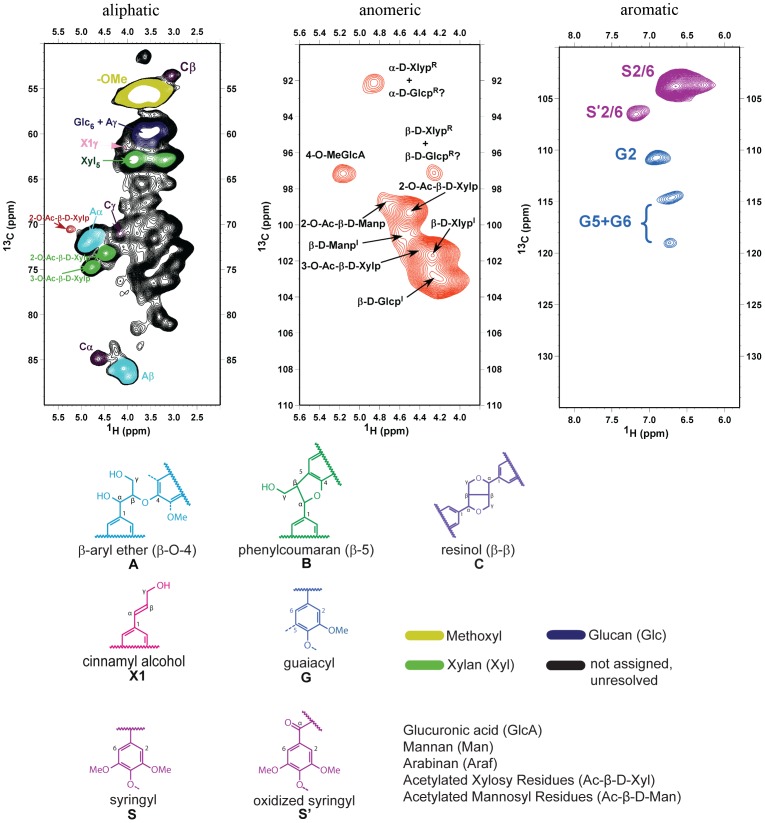
2D-NMR HSQC spectra of nonderivatized *E. globulus* cell wall separated into aliphatic, anomeric and aromatic regions. Color-coding in HSQC spectrum corresponding to known lignin substructures and polysaccharide units commonly found in plant cell walls. The main structures observed in the aliphatic region are: β-aryl ether units (A), phenylcoumaran units (B), resinol units (C), xylan (Xyl), acetylated xylosyl residues (2-O-Ac-β-D-Xylp and 3-O-Ac-β-D-Xylp), 2-acetylated mannosyl residues (2-O-Ac-β-D-Manp), and glucan (Glc6). The main structures observed in the anomeric region are: cellulose (β-D-Glcp), xylan (β-D-Xylp), acetylated xylosyl residues(2-O-Ac-β-D-Xylp and 3-O-Ac-β-D-Xylp), mannan (β-D-Manp), acetylated mannosyl residues (2-O-Ac-β-D-Manp), arabinan (β-D-Araf) and 4-O-methyl-α-D-glucuronic acid (4-O-MeGlcA). The main structures observed in the aromatic regions are: syringyl (S), oxidized syringyl (Ś) and guaiacyl (G) units.

2D-NMR of gels formed from the swelling of cryo-milled biomass samples in DMSO-d6 have been shown to enable the analysis of the cell wall without significantly altering its structure [30], [31], [32], [33], [34], [35]. This technique has made possible the relatively fast and detailed identification of certain lignin and polysaccharide monomers in the plant cell wall and their linkages in a comprehensive manner. The HSQC (1-bond ^13^C–^1^H correlation) spectra of *E. globulus* shows a representative spectrum of the plant-cell walls investigated in this study, with the common structures corresponding to the color-coded structures (Figure 1). The high-resolution spectra allow us to identify the majority of the peaks using published literature [18], [30], [31], [32], [33], [35], [36], [37]. Different lignin side chain and lignin unit correlations are readily distinguished in the aliphatic-oxygenated and aromatic regions of the HSQC spectra (Figure 1, aliphatic and aromatic). The 2D-NMR HSQC spectra of all other species can be found in Figure S1.

Aliphatic chemical-shift correlations for polysaccharides in the HSQC spectrum were strong and easily identified (Figure 1, anomeric): xylan (Xyl2: H2/C2, 3.01/72.6 ppm; Xyl3: H3/C3, 3.20/73.8 ppm; Xyl4: H4/C4, 3.52/75.4 ppm and Xyl5: H5/C5, 3.18–3.88/62.9 ppm), 2-acetylated xylan (2-O-Ac-β-D-Xylp: H2/C2, 4.49/73.2 ppm), 3-acetylated xylan (3-O-Ac-β-D-Xylp: H3/C3, 4.77/74.7 ppm), 2-acetylated mannan (2-O-Ac-β-D-Manp: H2/C2, 5.27/70.5 ppm), and glucan (Glc6: H6/C6, 3.55–59.9 ppm). Anomeric correlations for polysaccharides are found in the 3.8–5.8/90–110 ppm region and were identified using provisional assignments from published literature [30], with the peaks in this region being less well defined (Figure 1, anomeric). Cellulose, although the most abundant polysaccharide, is underrepresented in this region and in solution state-NMR in general, due to its highly crystalline structure and consequent poor swelling characteristics [30], [31], [32]. Chemical shifts assigned in this region are as follows: cellulose (β-D-Glcp^I^: 4.19/103.0 ppm), xylan (β-D-Xylp^I^: 4.27/102.8 ppm), acetylated xylosyl residues (2-O-Ac-β-D-Xylp: 4.50/99.3 ppm and 3-O-Ac-β-D-Xylp: 4.27/101.7 ppm), mannan (β-D-Manp^I^: 4.51/100.4 ppm), acetylated mannosyl residue (2-O-Ac-β-D-Manp: 4.68/98.7 ppm), arabinan (β-D-Araf^I^: 5.14/102.5 ppm), 4-O-methyl-α-D-glucuronic acid (4-O-MeGlcA: 5.15/97.1), reducing end of α-D-Glcp (α-D-Glcp^R^5.02/92.5 ppm), reducing end of α-D-Xylp (α-D-Xyl^R^ 4.86/92.1 pm) and reducing end of β-D-Xylp(β-D-Xylp^R^: H1/C1, 4.25/97.1 ppm).

The presence of strong acetylated-xylose crosspeaks in the hemicellulose is in agreement with previously published results [32], [38], [39], [40]. Strong acetate methyl peaks were observed at 2.00/20.7 ppm [31], [32]. These cross peaks are attributed to acetylated hemicelluloses as no cross peaks belonging to acetylated lignins were observed in the spectra of any species [31], [32]. There were also several cross peaks specific to individual species and tissue type. A strong cross peak at 5.08/100.0 ppm, assigned to α-L-fucopyranosyl [41] was found only in *A. mangium* heartwood and sapwood, *E. dunnii* sapwood and *E. urophylla* sapwood (Figure S1). The heartwood and sapwood of *E. pillularis* and *CCV* displayed notably strong correlations for 2-acetylated mannan residues (5.27/70.5 ppm and 4.68/98.7 ppm for the aliphatic and anomeric regions respectively). Only *A. magnium* heartwood lacked these cross peaks, and only a very weak signal was observed in its sapwood (Figure S1).

Lignin structures are present in both the aliphatic and aromatic region of the HSQC spectra. In the aliphatic region, the main lignin structures present are β-aryl ether units - A (Aα, 4.82/71.9 ppm; Aβ, 4.09/85.9 ppm), phenylcoumaran units - B (Bα, 5.43/86.9 ppm; Bβ, 3.45/53.1 ppm) and resinol units - C (Cα, 4.63/84.9 ppm; Cβ, 3.03/53.5 ppm; Cγ, 4.17/71.0 ppm) (Figure 1 anomeric). The relative presence of these units is calculated by using the integrated volume of Aα, Bα and Cα cross peaks. Among the species studied, *E. globulus* had the highest ratio of β-aryl ether units at 97%, while *E. pillularis* sapwood had the lowest at 91% (Table 1). *A. mangium* sapwood showed significantly higher amounts of phenylcoumaran linkages (Table 1). No phenylcoumaran units were observed in the heartwood of *E. globulus, E. dunnii*, and *CCV* or the sapwood of *CCV.* This is most likely due to lower ratios of guaiacyl units, derived from coniferyl alcohol, observed in these samples (Table 1). The absence of any dibenzodioxocin in all the species is also attributed to the lower levels of guaiacyl units [42]. Cinnamyl alcohol end-groups (X1, 4.07/61.4 ppm) were also observed in the aliphatic region of the HSQC spectra of all species studied (Figure 1 and Figure S1).

**Table 1:** The ratio of lignin substructures and crystallinity index of heartwood (HW) and sapwood (SW) samples.

	A:C:B	S:S′	S:G	CrI	CrI
				(height)	(area)
*Acacia mangium* HW	96∶3∶1	91∶9	57∶43	54	49
*CCV* HW	93∶7∶0	92∶8	95∶5	48	49
*E. dunnii* HW	94∶6∶0	91∶9	97∶3	46	40
*E. globulus* HW	97∶3∶0	94∶6	90∶10	51	29
*E. urophylla* HW	94∶5∶1	93∶7	84∶16	51	30
*E. urophylla* X *grandis* HW	95∶4∶1	92∶8	88∶12	44	41
*E. pillularis* HW	94∶5∶1	90∶10	86∶14	49	31
					
*Acacia mangium* SW	93∶3∶4	99∶1	44∶56	49	43
*CCV* SW	93∶6∶:1	91∶9	92∶8	47	40
*E. dunnii* SW	94∶6∶1	97∶3	85∶14	47	32
*E. globulus* SW	97∶3∶:0	86∶14	96∶4	54	47
*E. urophylla* SW	94∶5∶1	91∶9	76∶24	48	38
*E. urophylla* X *grandis* SW	96∶3∶1	92∶8	82∶18	52	43
*E. pilullaris* SW	91∶7∶2	88∶12	78∶22	56	40

The relative abundance of different inter-lignin linkages was determined by the integration of corresponding cross peaks in the HSQC spectra. Crystallinity index (CrI) of all samples was calculated using both the height of the XRD patterns or the area underneath. A (β-aryl ether); B (phenylcoumaran); C (resinol); S (syringyl); S′ (oxidized syringyl); G (guaiacyl).

The cross peaks observed in the aromatic region of the HSQC spectra belong to the aromatic rings of lignin monomers (Figure 1, aromatic). Strong S2/6 correlations for syringyl (S) and oxidized syringyl (Ś) units are observed at 6.66/103.8 ppm and 7.19/106.5 ppm respectively. For guaiacyl (G) units, G2 correlations are observed at 6.90/110.7 ppm and correlations for G5/6 are observed at 6.70/114.6 ppm and 6.76/119.02 ppm. No correlations for p-hydroxyphenyl (H) were detected in the HSQC spectra of any species. The S:S′ and S:G ratios (Table 1) were calculated based on the integration of the corresponding cross peaks. The sapwoods contained a higher variability in the relative abundance of oxidized syringyl (S′) units, which ranged from 1% (*A. mangium*) to 14% (*E. globules*). While the presence of these oxidized S′ units in *Eucalyptus* spp. has previously been suggested, it has also been suggested that this might be an artifact of the milling process [32], [34]. However, all the samples in this study were cryo-milled under the same conditions, suggesting that the variation is due to the differences in the composition of the plant cell wall. Syringyl (S and S′) and guaiacyl cross peaks were integrated to calculate S:G ratios (Table 1). The S:G ratios varied significantly, from 97∶3 for *E. dunnii* heartwood to 44∶56 for *A. mangium* heartwood, supporting previously published findings [32], [34], [43].

### Analysis by X-ray Diffraction

**Figure 2:**
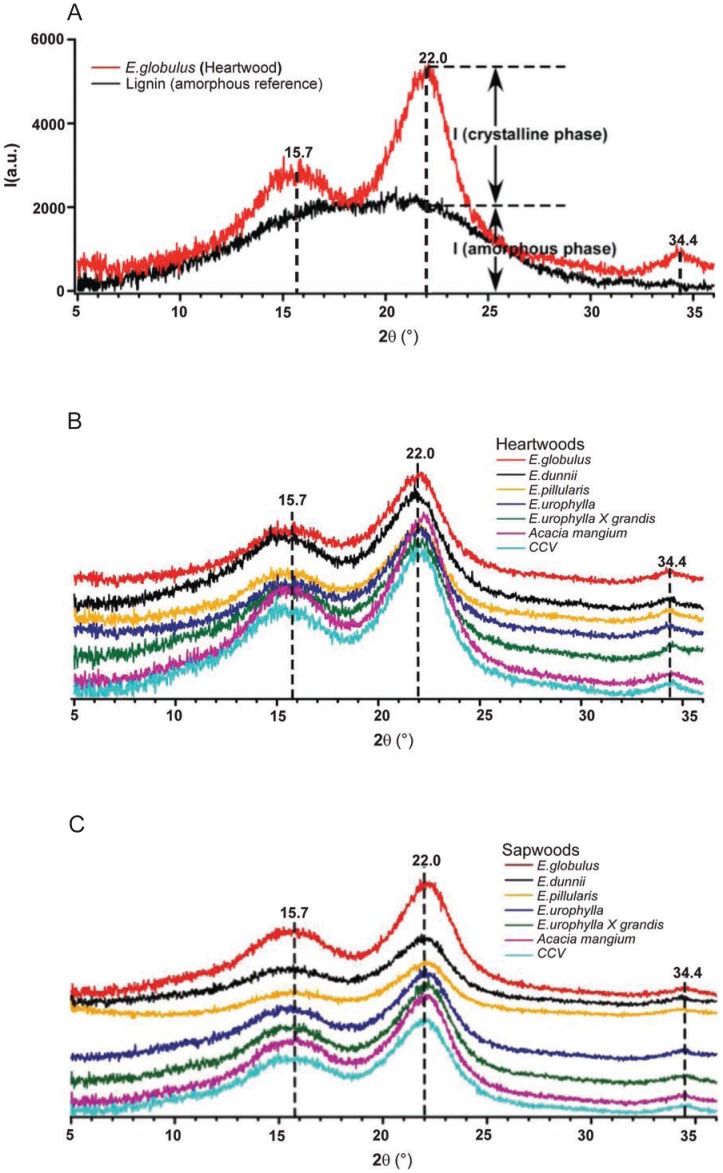
X-ray diffractograms of heartwood and sapwood samples. (A) Example of eucalyptus species (*E. globulus*) and reference (lignin) x-ray diffractogram to determine amorphous and crystalline phase. X-ray diffractograms of heartwoods (B) and sapwoods (C) of all hardwood species.

The crystalline nature of cellulose contributes to biomass recalcitrance by reducing the rate of enzymatic hydrolysis of *β*(1→4) linked D-glucosyl residues in the native cellulose chain through limiting the surface area for the reaction. The relative amounts of crystalline material in cellulose are defined by the crystallinity index that can be readily determined using X-ray diffraction (Figure 2A). XRD patterns for all the biomass samples were given in Figure 2B and 2C. There are three peaks at 15.7°, 22.0° and 34.4°, which are consistent with cellulose I lattice. The broad peak at 16.3° is a composite from several peaks and the peak at 22.0° corresponds to the distance between hydrogen-bonded sheets [18], [44]. The small peak at 34.4° corresponds to ¼ of the length of one cellobiose (1.036 nm) [45]. The calculated crystallinity based on the Segal method (peak height) of samples varied from 44 to 56% while that obtained applying the Ruland-Vonk method (peak area) was between 29 and 49 (Table 1). Both techniques require the baseline subtraction of an amorphous reference. The results obtained from the latter method are in closer agreement with the cellulose contents between 33 and 43% observed in the samples studied. Heartwood samples of *A. mangium* and *CCV* possessed the highest crystallinity while the sapwoods of *E. dunnii*, *E. pillularis,* and *E. urophylla* appear to have the lowest. The lack of a systematic trend within the two methods of calculating crystalinity points the need to confirm these findings using alternative methods such as solid state ^13^C NMR, FTIR and Raman. This will enable the development of more robust XRD correlations for these types of samples [46].

### Biochemical Composition of Extracted Biomass Samples

To provide a more detailed characterization of the biomass samples, total carbohydrate, lignin, cellulose and matrix polysaccharide contents were measured. Total carbohydrate and lignin content of all samples was obtained using two-step H_2_SO_4_ hydrolysis (Table 2). *CCV* and *A. mangium* were found to have the highest total-carbohydrate content in the heartwood at approximately 58%, with the heartwood content of *E. globulus* also being within experimental error. The other four-eucalypt species, *E. dunnii, E. pillularis, E. urophylla* and *E. urophylla X grandis* had similar carbohydrate contents at approximately 50%. Sapwood showed a smaller variation in total carbohydrates, but also displayed similar groupings, at 55% for *A. magnium*.*CCV* and *E. globulus*, and 52% for the other eucalypt species. These values are lower than those found in the literature, with the total carbohydrate composition of *E. globulus* typically being around 67% [9], [12], [13], [21], [22], [43], [47]. Approximately 20% of each sample was unaccounted for in the total mass closure. This is most likely due to sugars degrading under acidic conditions, not measured due to incomplete hydrolysis, and lignin estimations from the two-step H_2_SO_4_ protocol. Acetate, which typically comprises 3.5%^w^/_w_ of *E. globulus* [38], [39], [40], and some pectin-derived sugars were also not measured.

**Table 2:** Biochemical composition of extracted biomass samples of heartwood (HW) and sapwood (SW) as determined by two stage acid hydrolysis.

	Total carbohydrate	Lignin: acid insoluble	Lignin: acid soluble	Total lignin	Total (mass closure)
	(%)	(%)	(%)	(%)	(%)
*Acacia mangium* HW	57.9±5.9	27.1±0.8	1.5±0.6	28.6±1.0	86.5±6.0
*CCV* HW	58.9±2.0	21.6±1.2	3.4±0.6	25.0±1.4	83.9±2.4
*E. dunnii* HW	48.8±1.3	25.8±0.9	3.6±0.8	29.4±1.5	78.2±1.8
*E. globulus* HW	55.3±1.8	23.5±1.3	3.8±0.8	27.2±1.0	82.5±2.3
*E. urophylla* HW	50.4±2.3	28.7±0.5	2.8±0.6	31.5±1.1	81.9±2.5
*E. urophylla* X *grandis* HW	51.0±2.1	27.5±0.7	3.0±0.8	30.5±0.9	81.5±2.3
*E. pillularis* HW	49.6±3.6	27.5±0.9	2.9±0.7	30.4±1.1	80.0±3.8
					
*Acacia mangium* SW	55.5±2.8	28.7±1.3	1.1±0.2	29.8±1.3	85.3±3.0
*CCV* SW	55.8±2.6	26.5±1.6	2.8±0.6	29.3±1.7	85.1±3.1
*E. dunnii* SW	52.2±2.6	24.2±0.6	3.7±0.0	27.9±0.7	80.1±2.7
*E. globulus* SW	54.4±4.2	21.2±1.1	3.7±1.1	24.9±1.6	79.2±4.5
*E. urophylla* SW	51.5±2.0	25.8±0.8	2.5±0.1	28.3±0.6	79.8±2.1
*E. urophylla* X *grandis* SW	52.8±3.2	26.7±1.2	2.9±0.5	29.5±0.9	82.3±3.5
*E. pillularis* SW	51.3±1.1	28.7±0.7	2.6±0.1	31.3±1.1	82.6±1.3

Values are presented as a percentage weight of the starting extracted biomass. Mass closure is the percentage mass total of total carbohydrates and total lignin.

Total lignin content measured varied between 25% for *E. globulus* sapwood and 31%, for *E. urophylla* heartwood among all the samples analyzed (Table 2). The acid insoluble and the total lignin content of *CCV* heartwood and *E. globulus* sapwood were lower compared to other species, and *A. magnium* had the lowest levels of the acid soluble lignin. Overall, the variations in lignin content were similar between heartwoods and the sapwoods.

The cellulose content of hardwoods is a key attribute for potential utilization for biofuels production. As a proportion of total measured carbohydrate, glucose derived from cellulose in both heartwood and sapwood ranged from 68% (*E. globulus,* HW) to 78% (*E. urophylla* X *grandis* SW). Generally, the cellulose content of sapwoods was higher than that of heartwoods in all species analyzed, an observation that has previously described for a number of hardwood species [47].

Analysis of the matrix polysaccharides in each extracted biomass sample consistently showed the presence of rhamnose, fucose, arabinose xylose, mannose, galactose, glucose and galacturonic acid (Table 3). Glucuronic acid levels in the samples were negligible with only detectable amounts in about half the samples. Xylose was the most predominant monosaccharide in all samples ranging from 15% (*E. dunnii* SW) and 24% (*E. urophylla* HW). Of the other non-cellulosic derived sugar, galacturonic acid, levels were higher in heartwood samples when compared to sapwood, with the exception of *A. mangium*, indicating slightly higher levels of pectin (e.g. polygalacturonan) in this tissue. While the amount of mannose was relatively low in all samples, *CCV* contained a higher proportion in both wood types relative to the other species. This may indicate increased levels of some hemicelluloses such as glucomannan. Other minor sugars such as non-cellulosic glucose, rhamnose and arabinose have been attributed to short chained polysaccharide fragments that are chemically linked to the heteroxylan backbone [23], [40].

**Table 3:** Detailed carbohydrate biochemical composition of extracted biomass samples of heartwood (HW) and sapwood (SW) as determined by HPAEC and Updegraff.

	Average Fucose (%)	Average Rhamnose (%)	Average Arabinose (%)	Average Galactose (%)	Average Glucose (%)	Average Xylose (%)	Average Mannose (%)	Average Galacturonic Acid (%)	Average Glucuronic acid (%)	Average Cellulose (%)
***Acacia mangium*** ** HW**	0.05±0.01	0.53±0.01	0.54±0.06	1.45±0.12	1.71±0.12	19.68±0.28	0.27±0.02	2.88±0.06	NA	72.90±3.28
***CCV*** ** HW**	0.03±0.01	0.74±0.03	0.36±0.01	1.18±0.11	2.07±0.20	23.00±0.43	1.14±0.05	3.30±0.08	NA	68.18±0.83
***E. dunnii*** ** HW**	0.06±0.01	0.91±0.03	0.64±0.04	1.40±0.02	1.85±0.06	19.34±0.20	0.54±0.02	4.00±0.07	0.01±0.02	71.24±3.76
***E. globulus*** ** HW**	0.04±0.01	0.77±0.02	0.43±0.04	1.47±0.09	2.05±0.09	22.99±1.48	0.50±0.06	3.23±0.15	0.02±0.02	68.51±7.34
***E. urophylla*** ** HW**	0.04±0.01	0.57±0.04	0.30±0.05	1.97±0.09	1.73±0.15	23.75±1.11	0.36±0.01	3.59±0.18	0.01±0.02	67.67±12.77
***E. urophylla*** ** X ** ***grandis*** ** HW**	0.03±0.01	0.74±0.05	0.29±0.02	2.53±0.23	1.80±0.22	19.01±0.64	0.25±0.04	3.44±0.17	0.01±0.02	71.90±3.84
***E. Pillularis*** ** HW**	0.03±0.01	0.47±0.01	0.24±0.01	1.65±0.03	1.82±0.16	18.85±0.16	0.28±0.04	3.18±0.14	NA	73.48±2.33
										
***Acacia mangium*** ** SW**	0.04±0.01	0.54±0.03	0.44±0.03	1.05±0.02	3.04±0.24	22.20±0.74	0.37±0.10	2.85±0.14	NA	69.48±9.25
***CCV*** ** SW**	0.04±0.01	0.59±0.02	0.37±0.02	0.67±0.03	2.26±0.04	19.38±0.40	1.43±1.10	2.58±0.06	NA	72.69±5.42
***E. dunnii*** ** SW**	0.03±0.01	0.56±0.02	0.46±0.03	1.39±0.06	2.58±0.11	15.17±0.36	0.44±2.10	2.73±0.14	NA	76.65±1.90
***E. globulus*** ** SW**	0.02±0.01	0.58±0.03	0.32±0.03	1.03±0.03	3.00±0.23	17.27±0.84	0.29±3.10	2.59±0.07	0.01±0.01	74.89±1.14
***E. urophylla*** ** SW**	0.02±0.01	0.34±0.02	0.25±0.02	1.49±0.04	1.99±0.23	18.48±0.54	0.68±4.10	2.57±0.10	NA	74.17±3.70
***E. urophylla*** ** X ** ***grandis*** ** SW**	0.03±0.01	0.41±0.02	0.26±0.01	1.46±0.10	1.71±0.03	15.18±0.70	0.43±5.10	2.70±0.30	NA	77.83±1.92
***E. Pillularis*** ** SW**	0.02±0.01	0.32±0.01	0.15±0.01	1.03±0.03	2.00±0.07	17.60±0.43	0.21±6.10	2.34±0.01	NA	76.33±5.53

Values are presented as a percentage weight of the AIR mass.

### Analysis of Samples by Fourier Transform Near Infrared Spectroscopy

**Figure 3:**
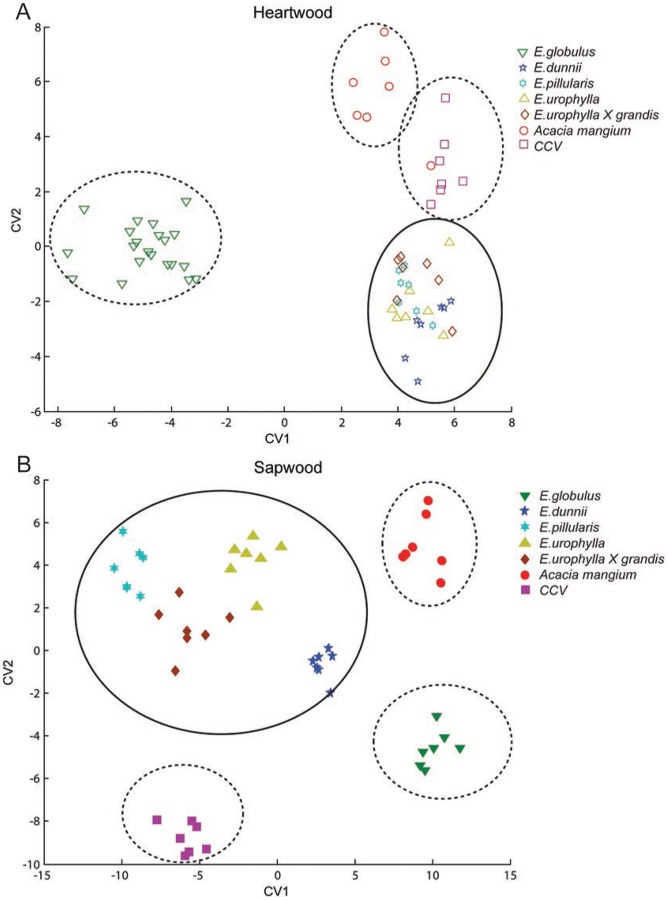
Canonical variate analysis of FT-NIR spectra of heartwood (A) and sapwood (B) demonstrating clustering of species into district groups.

FT-NIR spectroscopy has been used for the rapid characterization and classification of plant material [48], [49], [50], [51]. In contrast to mid infrared, the NIR region (12,000 to 4,000 cm^−1^) does not reveal discrete signature peaks, but it excites several harmonic overtones of methyl, aromatic CH-OH, with minor features in methoxy and carbonyl CH bonds, generating spectra that have no easily distinguishing chemical features [52]. However, by using multivariate analysis, it is possible to identify samples whose spectra differ from the average population [53], [54], [55]. Here, Canonical Variant Analysis (CVA) was employed to compare inter-group (species) variability relative to intra-group (biological and technical) variability [27]. CVA showed that the heartwoods of *A. mangium*, *CCV* and *E. globulus* appear to be separate from the other species (Figure 3A). For sapwood, the clustering was more distinct, with points from each species grouping discretely together, and *A. mangium*, *E. dunnii*, *E. globulus* and *CCV* appearing as outlier populations (Figure 3B). Comparison to compositional data show that for heartwoods, formation of outlier groups could be attributed to high carbohydrate and low lignin contents (Table 1). Sapwood outliers corresponded also to high carbohydrate content and cellulose crystallinity calculated via Ruland-Vonk method (peak area). Though FT-NIR does not generate detailed chemical or structural information, it can be used in a screening manner without any additional biochemical information. Further sampling and correlation with other physical and chemical properties should help to identify and confirm the factors causing the out-grouping of these species, and enable the use of FT-NIR for the rapid identification of eucalypts that are better candidates for biofuels production.

### Conclusions

Samples showing differences in composition are likely to be of great importance for the development of future biofuels. For this reason, we have characterized widely utilized eucalypt species and a commonly planted tropical wattle. The data demonstrate significant diversity in composition amongst these species that will be important in selecting candidates for the development of feedstocks for biofuels. In general, the structural and biochemical composition were remarkably similar between all species. However, subtle differences were observed especially in the case of *A. mangium* heartwood where no acetylated mannan cross peaks were observed and the L-fucopyranosyl cross peaks were more prominent in the NMR spectra relative to other samples and S:G ratio was the lowest. We could also demonstrate that FT-IR could be a valuable/robust technique for quick characterization of such samples. Further screening of eucalypts and other potential feedstock species will be useful in developing more robust correlative relationships between rapid screening techniques such as FT-NIR and the suitability of a species for biofuel or pulp production.

## Supporting Information

Figure S1 **Complete 2D-NMR HSQC spectra of non derivatized cell wall material and key for all the species analyzed in this study (** ***Eucalyptus dunnii*** **,** ***Eucalyptus globulus*** **,** ***Eucalyptus pillularis*** **,** ***Eucalyptus urophylla*** **,** ***Eucalyptus urophylla*** **-** ***Eucalyptus grandis*** **cross,** ***Corymbia citriodora*** **subsp.** ***variegata*** **(** ***CCV*** **) and** ***Acacia mangium*** **) for both heartwood and sapwood samples.**
(TIF)

Table S1 **Moisture content of samples measured in triplicate. Values are presented as a percentage weight of the starting extracted biomass.**
(DOCX)
