# Supplementary figures and images for: Structural and Chemical Characterization of Hardwood from Tree Species with Applications as Bioenergy Feedstocks
Source: PLoS One. 2012 Dec 28;7(12):e52820. doi: 10.1371/journal.pone.0052820 (PMC3532498; doi:10.1371/journal.pone.0052820)

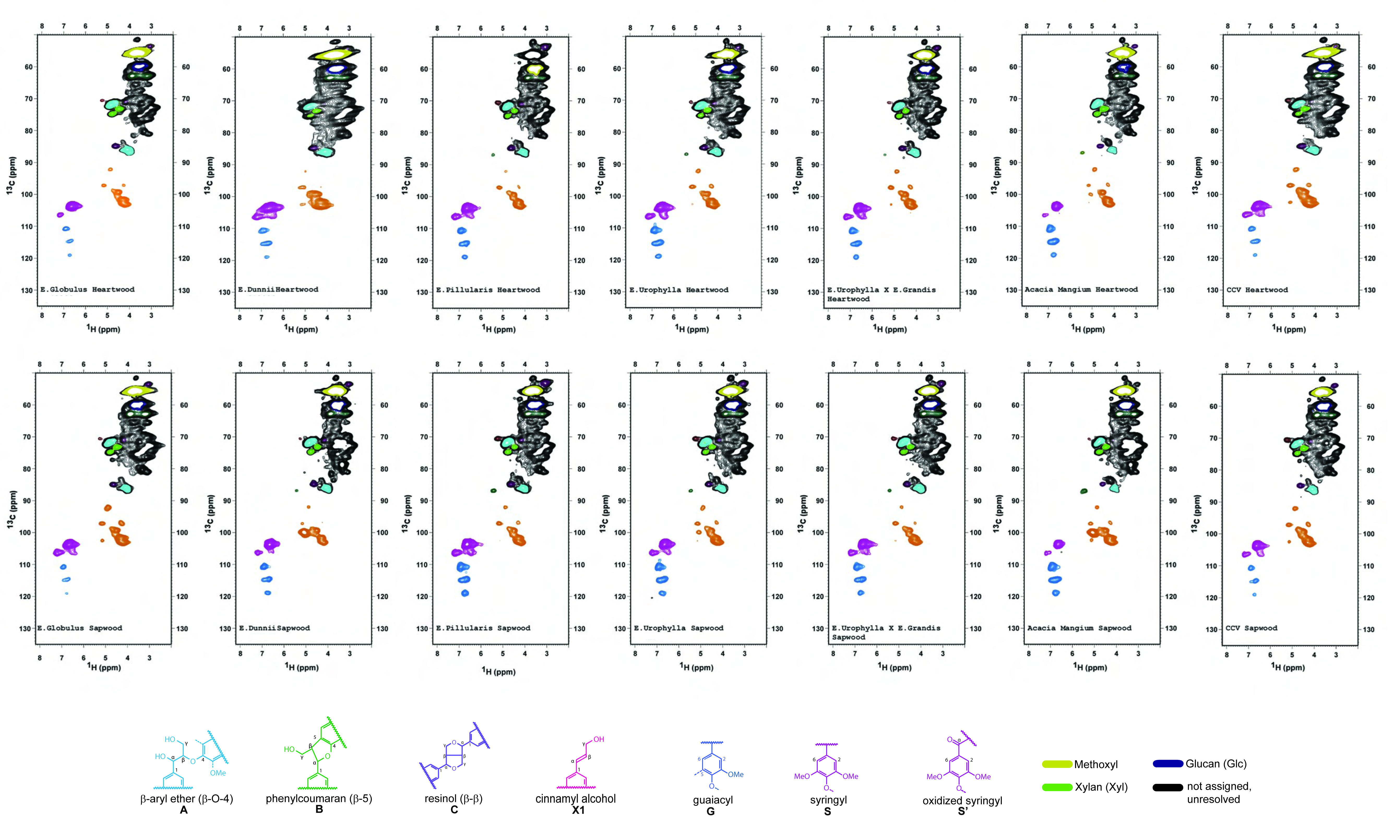

Supplement: Figure S1 — Complete 2D-NMR HSQC spectra of non derivatized cell wall material and key for all the species analyzed in this study ( Eucalyptus dunnii , Eucalyptus globulus , Eucalyptus pillularis , Eucalyptus urophylla , Eucalyptus urophylla - Eucalyptus grandis cross, Corymbia citriodora subsp. variegata ( CCV ) and Acacia mangium ) for both heartwood and sapwood samples. (TIF) [file pone.0052820.s001.tif]
