# Supplementary material for: Structural and Chemical Characterization of Hardwood from Tree Species with Applications as Bioenergy Feedstocks
Source: PLoS One. 2012 Dec 28;7(12):e52820. doi: 10.1371/journal.pone.0052820 (PMC3532498; doi:10.1371/journal.pone.0052820)
Supplement: Table S1 — Moisture content of samples measured in triplicate. Values are presented as a percentage weight of the starting extracted biomass. (DOCX) [file pone.0052820.s002.docx]

|  | **Moisture Content (%)** |
| --- | --- |
| ***Acacia mangium* HW** | 91.4 ± 0.3 |
| ***CCV* HW** | 92.6 ± 0.1 |
| ***E. dunnii* HW** | 92.6 ± 0.2 |
| ***E. globulus* HW** | 91.8 ± 0.2 |
| ***E. urophylla* HW** | 93.0 ± 0.3 |
| ***E. urophylla* X *grandis* HW** | 91.7 ± 0.1 |
| ***E. pillularis* HW** | 92.7 ± 0.1 |
|  |  |
| ***Acacia mangium* SW** | 92.1 ± 0.3 |
| ***CCV* SW** | 92.0 ± 0.2 |
| ***E. dunnii* SW** | 91.8 ± 0.3 |
| ***E. globulus* SW** | 91.7 ± 0.3 |
| ***E. urophylla* SW** | 92.6 ± 0.3 |
| ***E. urophylla* X *grandis* SW** | 91.4 ± 0.2 |
| ***E. pillularis* SW** | 92.0 ± 0.7 |

**Table S1**. Moisture content of samples measured in triplicate. Values are presented as a percentage weight of the starting extracted biomass.
